# Aseptic Meningitis and Depression: The Neuropsychiatric Manifestations of a Patient with Systemic Lupus Erythematosus

**DOI:** 10.7759/cureus.5424

**Published:** 2019-08-19

**Authors:** Ivania T Irby, Patrycia Leja, Donald Manning, Kaustubh Limaye, Sourabh Lahoti

**Affiliations:** 1 Psychiatry, Coliseum Medical Center, Macon, USA; 2 Miscellaneous, Carl Vinson Veterans Affairs Medical Center, Dublin, USA; 3 Neurology, University of Iowa, Iowa City, USA; 4 Neurology, Coliseum Medical Center, Macon, USA

**Keywords:** aseptic, neuropsychiatric, meningitis, lupus, depression, encephalitis, npsle, neuroimaging, vasculitis, sinusitis

## Abstract

Aseptic meningitis as an initial and isolated manifestation of systemic lupus erythematosus (SLE) is extremely rare. About a third of patients with SLE have neuropsychiatric manifestations; however, less than 2% develop aseptic meningitis. Therefore, SLE as a cause of aseptic meningitis is commonly overlooked and leads to delayed or even missed diagnosis. We report a case of aseptic meningitis that was later discovered to be SLE and where instituting appropriate treatment led to clinical improvement.

## Introduction

The neuropsychiatric (NP) manifestations of systemic lupus erythematosus (SLE) includes disorders related to mood and emotions, memory impairment and general cognitive decline, among others. Less than two percent develop aseptic meningitis [1-2]. Therefore, SLE as a cause of aseptic meningitis is sometimes ignored and leads to delayed or even missed diagnosis as well as unnecessary testing and imaging studies.

Neuropsychiatric manifestations of SLE (NPSLE) are found in approximately 14%-80% of all adult patients diagnosed with SLE. This wide prevalence of NP events is due in part to methodologic differences between studies. Those manifestations can mimic symptoms related to current illness, medication use, and functional disturbances. It has been reported that patients with NPSLE have an increased risk of death and morbidity [3].

Primary NPSLE had been defined based on the presentation of symptoms either as focal form related to lupus associated coagulopathies that include stroke or focal seizures or as diffuse manifestations in which clinical presentation varies between patients, and may include mood symptoms such as depression, anxiety and general cognitive impairment [4]. Several efforts have been made in order to determine the diagnostic criteria for NPSLE, although specific testing does not exist. The most accepted criteria was written in 1999 by the American College of Rheumatology. A high level of clinical suspicion, laboratory, and neuroimaging studies to support diagnosis and exclude related pathologies are necessary to support a diagnosis [5].

There has been a great amount of recent effort in neuropsychiatric research to identify the exact mechanism of the NPSLE to address behavior and cognition as describe in this review [3-4].

We report a case of aseptic meningitis that was initially diagnosed at an urgent care center as sinusitis. When the patient was later brought to the emergency department, she presented with complex neurological symptoms and several possible diagnoses were considered. After differential diagnoses were ruled out by numerous laboratory testing and imaging studies, SLE was revealed. Instituting a multimodal treatment of steroids and acute rehabilitation led to clinical improvement.

## Case presentation

A 34-year-old African American female was brought to the emergency department with complaints of fever, headaches, and weakness in both arms and legs over the past five days. She had acute onset of severe, holocranial headache before arrival which was associated with nausea and nasal congestion. She was diagnosed with sinusitis at an urgent care clinic and prescribed oral antibiotics and analgesics. These medications did not provide any relief. The patient developed nausea, photophobia, and weakness of both arms and legs two days later. Her sister eventually brought her to the emergency room for sleepiness along with weakness of all the limbs. She was febrile, tachycardic, lethargic, but able to follow commands and answer simple questions. On neurological examination, she had photophobia, neck stiffness and effort-dependent weakness in her extremities along with bilateral hearing loss on finger rub test. Infectious meningitis was considered followed by encephalitis, subarachnoid hemorrhage, reversible cerebral vasoconstriction syndrome (RCVS) and vasculitis. Computed tomography (CT) of the head was obtained which was unremarkable. Cerebrospinal fluid (CSF) analysis showed pleocytosis (123 cells/cu.mm) with increased protein (125 mg/dl), normal glucose (52 mg/dl) and a significant red blood cell count (4620/cu.mm). Concern for traumatic tap was raised. Repeat tap under fluoroscopic guidance showed similar results. Viral meningitis was suspected. CSF culture and Gram stain were requested as well as HSV PCR and therapy with acyclovir was started. The results of these tests were negative. Magnetic resonance imaging (MRI) of the head without contrast did not show any abnormality. There was no improvement in her symptoms even after five days of antimicrobial and antiviral therapy. She developed apathy, markedly diminished interest, psychomotor retardation and decreased appetite, suggestive of depressed mood. Additional CSF and serologic tests to investigate for autoimmune meningitis were requested. Positive report for anti-Smith antibodies (>8.0 reference: 0.0-0.9) and Ribonucleoprotein antibodies (7.7 reference: 0.0-0.9) indicated SLE. Collateral information was obtained from her family and it was discovered she had been diagnosed with SLE 12 years ago and was on steroids for two years.

She then stopped seeing her rheumatologist and discontinued her medications. She was asymptomatic in the interim period until this hospital admission. She did not have any psychiatric history. Treatment with dexamethasone 10 mg IV once was started followed by dexamethasone 4 mg PO QID for the first two days and then switched to prednisone (1 mg/kg) 70 mg qd. Evaluation for cerebral vasculitis was needed because of the findings of red blood cells in the CSF and the strong association of SLE with vasculitis. Catheter cerebral angiogram was done showing normal cerebral vascularity. However, this angiogram was performed three days after starting high-dose steroids. By days 3-5 of the initiation of steroids, before discharge, she was alert, oriented and interacting. There was a substantial improvement in her thought process, psychomotor activity, and appetite. However, she continued to have generalized muscular weakness. Therefore, she was discharged to an acute rehabilitation unit for deconditioning.

## Discussion

Aseptic meningitis has been described as one of the 19 NPSLE and can be one of the first signs of SLE [5]. It can lead to an elevated cell count in CSF with predominantly mononuclear cells, an elevated protein level, and normal glucose. However viral, fungal and parasitic infections, tuberculosis, partially treated bacterial meningitis and neoplastic meningitis all produce similar CSF pattern [2,6]. Viral meningitis was the initial diagnostic consideration because of its higher prevalence compared to aseptic meningitis, and since no improvement was seen we expanded our search for other possible causes and subsequent discovery of SLE.

The precise pathophysiology of SLE-associated aseptic meningitis remains unknown. The mechanism of SLE meningitis is hypothesized to be consequent to DNA-anti-DNA immune complexes that are dispersed and deposited in the choroidal plexus and to the low complement levels in CSF during active CNS disease [4,7]. Another explanation is SLE’s association with vasculitis through autoreactive antibodies [2,4]. In some cases, drug-induced aseptic meningitis has been suggested. Ironically, drugs used for the treatment of SLE, such as nonsteroidal anti-inflammatory drugs (NSAIDs), antimalarials (hydroxychloroquine sulfate), intravenous immunoglobulin (IVIG) and OKT3 monoclonal antibodies are the notable culprits [3]. Our patient, however, was not receiving any treatment for SLE.

The most accepted diagnostic criteria for NPSLE is by the American College of Rheumatology, published in 1999. They recommend confirming the diagnosis of lupus by taking a detailed history and physical examination, excluding systematic illnesses and medications, and requesting the appropriate tests based on the findings [5]. The most frequent manifestations are headaches (28.3%), mood disorders (20.7%) and cognitive dysfunction (19.7%). Some reports suggest up to 40% of neuropsychiatric symptoms appear during the first year of SLE diagnosis [8].

Depression is the most common mood disorder in NPSLE. Depression is linked to several factors, including the use of steroids for treatment, diagnosis of SLE and the disease activity. There is a strong association of depression and specific antibodies directed at ribosomal-P, N-methyl-d-aspartate (NMDA) receptor and other neuronal epitopes [9]. The mechanism is thought to be through neuronal apoptosis followed by breaching of the blood-brain barrier with lipopolysaccharide causing hippocampal neuron damage [4,9]. Our patient had signs that indicated depressed mood that responded satisfactorily to intravenous steroids and it was most likely due to uninhibited disease activity.

The diagnosis of neuropsychiatric lupus could be difficult in patients without a known history of SLE. In our case, the patient ignored the fact that she was diagnosed with SLE 12 years prior.

Neuroimaging modalities such as functional MRI, diffusion tensor imaging, positron emission tomography (PET) scan, and magnetic resonance spectroscopy (MRS) are promising tools to help identify the mechanism involved in NPSLE and the manifestations of cognitive dysfunction. Brain imaging studies can indicate which areas of the brain are affected by NPSLE. Yet, these modalities are not without limitations [3-4]. The most common findings are dilation of the ventricles, cortical atrophy infarcts, and white matter hyper-intense lesions. Meanwhile, functional imaging can reflect changes in perfusion, or hypometabolism and neuronal activity. Some of these studies quantify some brain biochemical compounds such as reduction in N-acetyl aspartate representing neuronal or axonal dysfunction or loss and can correlate with cerebral atrophy and neurocognitive dysfunction. Others have shown abnormal diffusivity consistent with inflammation and loss of white matter integrity and discernment between inflammatory and ischemic lesions [3].

Once the diagnosis of NPSLE has been established, the treatment is multimodal. Although there is still no evidence-based treatment approach, data support the safety of biological medication when classic treatment fails [1]. The European League Against Rheumatism (EULAR) created a treatment recommendation for SLE and NPSLE in 2010 [10]. The agents of choice include glucocorticoids and antimalarials for individuals afflicted with SLE with no major organ dysfunction. Immunosuppressive therapy is usually the last line for cases which no longer respond to first line agents or when steroid doses become too high. NPSLE is typically treated in the same manner as if the patient did not have SLE. For instance, a patient presenting with depressive symptoms may be prescribed a selective serotonin reuptake inhibitor (SSRI) or other first line options. When NPSLE symptoms are related to antiphospholipid antibodies such as ischemic stroke, anticoagulant therapy is recommended. Otherwise, if NPSLE symptoms are associated with an inflammatory condition such as aseptic meningitis, glucocorticoids alone or with immunosuppressive therapy is appropriate [10].

Only three medications are United States Food and Drug Administration (USFDA) approved for the treatment of SLE: glucocorticoids, aspirin, and hydroxychloroquine. Belimumab was recently approved by the USFDA in July 2017 [11]. The choice of the treatment for NPSLE in each patient should be individualized based on the clinical manifestations, severity of the disease and potential pathogenic mechanism [9].

## Conclusions

Aseptic meningitis can be one of the first signs of SLE. There is no specific test for cerebral SLE, therefore, to aid diagnosis of NPSLE: confirm the diagnosis of lupus; take a detailed history and physical examination; exclude systematic illness and medication; request the appropriate tests based on the findings. Neuroimaging modalities are promising tools to help identify the mechanism involved in NPSLE and the manifestations of cognitive dysfunction.

## References

[1] Hanly JG, Urowitz MB, Wallace DJ (2007). Neuropsychiatric events at the time of diagnosis of systemic lupus erythematosus: an international inception cohort study. Arthritis Rheum.

[2] Jennekens FG, Kater L (2002). The central nervous system in systemic lupus erythematosus. Part 1. Clinical syndromes: a literature investigation. Rheumatology.

[3] Popescu A, Kao AH (2011). Neuropsychiatric systemic lupus erythematosus. Curr Neuropharmacol.

[4] Stock AD, Gelb S, Pasternak O, Ben-Zvi A, Putterman C (2017). The blood brain barrier and neuropsychiatric lupus: new perspectives in light of advances in understanding the neuroimmune interface. Autoimmune Rev.

[5] Liang MH, Corzillius H, Bae SC (1999). The American College of Rheumatology nomenclature and case definitions for neuropsychiatric lupus syndromes. Arthritis Rheum.

[6] Campbell W (739-741). DeJong’s The Neurologic Examination.

[7] Canoso JJ, Cohen AS (1975). Aseptic meningitis in systemic lupus erythematosus. Arthritis Rheum.

[8] Unterman A, Nolte JE, Boaz M, Abady M, Shoenfeld Y, Zandman-Goddard G (2011). Neuropsychiatric syndromes in systemic lupus erythematosus: a meta-analysis. Semin Arthritis Rheum.

[9] Kivity S, Agmon-Levin N, Zandman-Goddard G, Chapman J, Shoenfeld Y (2015). Neuropsychiatric lupus: a mosaic of clinical presentations. BMC Med.

[10] Bertsias G, Ioannidis J, Aringer M (2010). EULAR recommendations for the management of systemic lupus erythematosus with neuropsychiatric manifestations: report of a task force of the EULAR standing committee for clinical affairs. Ann Rheum Dis.

[11] Drugs@FDA: FDA Approved Drug Products. (n.d.). Retrieved from. https://www.accessdata.fda.gov/scripts/cder/daf/index.cfm?event=overview.process&ApplNo=761043.

